# A novel data processing method CyC* for quantitative real time polymerase chain reaction minimizes cumulative error

**DOI:** 10.1371/journal.pone.0218159

**Published:** 2019-06-11

**Authors:** Linzhong Zhang, Rui Dong, Shu Wei, Han-Chen Zhou, Meng-Xian Zhang, Karthikeyan Alagarsamy

**Affiliations:** 1 State Key Laboratory of Tea Plant Biology and Utilization, Anhui Agricultural University, Hefei, Anhui, China; 2 School of Science, Anhui Agricultural University, Hefei, Anhui, China; 3 Tea Research Institution, Anhui Academy of Agricultural Sciences, Huangshan, Anhui, China; Defense Threat Reduction Agency, UNITED STATES

## Abstract

Quantitative real-time polymerase chain reaction (qPCR) is routinely conducted for DNA quantitative analysis using the cycle-threshold (*Ct*) method, which assumes uniform/optimum template amplification. In practice, amplification efficiencies vary from cycle to cycle in a PCR reaction, and often decline as the amplification proceeds, which results in substantial errors in measurement. This study reveals the cumulative error for quantification of initial template amounts, due to the difference between the assumed perfect amplification efficiency and actual one in each amplification cycle. The novel CyC* method involves determination of both the earliest amplification cycle detectable above background (“outlier” C*) and the amplification efficiency over the cycle range from C* to the next two amplification cycles; subsequent analysis allows the calculation of initial template amount with minimal cumulative error. Simulation tests indicated that the CyC* method resulted in significantly less variation in the predicted initial DNA level represented as fluorescence intensity *F*_0_ when the outlier cycle C^*^ was advanced to an earlier cycle. Performance comparison revealed that CyC* was better than the majority of 13 established qPCR data analysis methods in terms of bias, linearity, reproducibility, and resolution. Actual PCR test also suggested that relative expression levels of nine genes in tea leaves obtained using CyC* were much closer to the real value than those obtained with the conventional 2^-ΔΔCt^ method. Our data indicated that increasing the input of initial template was effective in advancing emergence of the earliest amplification cycle among the tested variants. A computer program (CyC* method) was compiled to perform the data processing. This novel method can minimize cumulative error over the amplification process, and thus, can improve qPCR analysis.

## Introduction

Quantitative real-time polymerase chain reaction (qPCR) employs fluorescent dyes such as SYBR Green or Taqman probe; these dyes intercalate into double strand DNA products to allow easy determination of amplified DNA amounts in each amplification cycle by detecting fluorescence intensity [1]. Because of its simplicity, efficiency and sensitivity [2], qPCR has become a routine technique in various biological studies and practical applications such as the noncoding small interfering RNA [3,4], differential gene expression [5,6], transgenic T-DNA tandem repeat analysis [7], virus titer evaluation [8] and diagnostic tools [9,10]. The two quantification methods often applied are absolute quantification and relative quantification. Absolute quantification is conducted based on an assumption that amplification efficiencies for both the target template and the standard template DNA used for calibration curve construction should be identical [11–13], and relative quantification determines relative transcript levels of a gene across multiple samples [12,14,15]. For a relative quantitative analysis, the comparative cycle-threshold (*Ct*) method [13] is widely accepted as a practical and feasible “golden method”. However, this method is based on the assumption that amplification efficiency for both target and reference genes is perfect (100%) or constant [12,15]. A slight PCR amplification efficiency decrease of about 4% could result in an error of up to 400% for a gene expression ratio [16].

The amplified products in the course of the reaction follow a kinetic time-discrete pattern. The amount of accumulated amplification products ($S_{C}$) is a function of the initial amount of DNA strands ($S_{0}$) and the amplification efficiency ($E_{i}$) after *C* cycles, and is described in Equation (Eq 1) [17]. The amplification kinetics gives the amount of fluorescent dye intercalated DNA template, which increases exponentially during cycling.

$$S_{C}=S_{0}\prod_{i=0}^{C}(1+E_{i})$$ (1)

In practice, the amplification efficiency ($E_{i}$) of a PCR reaction changes dynamically over the reaction course [18]. Earlier cycles result only in background fluorescence, and $E_{i}$ declines in later cycles [19–21]. Among the various possible reasons for inhibition of amplification [22], substrate depletion, inactivation of the polymerase enzyme, product inhibition and fractional re-annealing of the template strands are involved in the amplification saturation process [21]. In addition, primers length, amplificon sequence length, and their respective G+C contents were reported as the most significant factors affecting amplification efficiency [18].

Many efforts have been made to overcome these challenges to obtain a better quantification of the initial levels of DNA or RNA fragments using assumption-free methods [2,23–25]. For instances, the improved *Cy0* method was proposed to compensate for efficiency variation using the efficiency parameter estimated with the inflection point [2], Linear regression on the amplified product fluorescence data was also proposed as an assumption-free method for calculating the initial template amount [23]. An amplification kinetics study has revealed that the observed PCR efficiency values could be affected by the errors in determination of background fluorescence, and thus, are propagated exponentially in quantitation of initial template amount and relative abundance or ‘fold-change’ [26]. It is imperative to deal with such a problem for a more accurate quantitative analysis.

In this study, a cumulative error in the amplification process was revealed due to the difference between the actual template amount and the amount estimated based on the assumption of perfect amplification efficiency. In this study, the CyC* method was proposed as a novel approach based on determining the outlier cycle C*, which represents the initiation point of the amplification process to minimize cumulative error and improve the accuracy of gene expression analyses. The amount of initial template, amplified product size and primer-template mismatch numbers were also examined for improvement of accuracy, and software was compiled to provide a free and easy-to-use analysis application. Our data indicate that this method is feasible for conducting quantitative analysis of gene transcript levels.

## Materials and methods

### Nucleic acid samples

In this study, DNA segments used for quantification assays were either the plant genes or partial gene sequences present in pEASY-T1 plasmids (GenBank EU233623.1). The plasmids previously constructed in this lab each contained a PCR cloned gene of our interest, such as *GREEN FLUORESCENT PROTEIN* (*GFP*) (GenBank, U87973.1) (717 bp), *NEOMYCIN PHOSPHOTRANSFERASE* (*npt* II) (GenBank, ABW88015.1) present in the pEASY-T1. Isolation of these plasmids from *Escherichia coli* strain DH5α was performed with Axygen Plasmid Midi Kit (Corning China, Shanghai) using the manufacturer’s instructions. These plasmids were used to test the effects of initial template amounts, amplified product sizes, and primer mismatches on outlier cycle emergence in a PCR procedure. For plant gene transcript quantification, young leaves of cv. “Shu-cha Zao” (*Camellia sinensis*) were used to extract total RNA using RNAprep pure Plant Kit (TianGen Biotech., Beijing, China). Complimentary DNA (cDNA) was synthesized using 2 μg of total RNA and SuperScriptII reverse transcriptase (Invitrogen, Shanghai, China). Quality and quantity of DNA and RNA samples were determined using both agarose gel electrophoresis and the Nanodrop 2000 spectrophotometer (Thermo Fisher Scientific, Wilmington, DE, USA).

### Analyses of some PCR variants

For testing the effects of different factors on the emergence of PCR outliers, template amounts, amplification product sizes, and primer mismatches were examined under controlled reaction parameters. For the testing template input amount, a ten-fold serial dilution of plasmid pEASY-GFP was prepared with concentrations of 0.001–10 ng for application in qPCR reaction mixtures. For the different amplicon size test, the primer pairs (**S1 Table**) were designed with the same parameters such as oligo length and Tm values using Primer 5 program to generate a series of increasingly large products (116 bp, 347 bp, 747 bp, and 1349 bp). For the primer mismatch experiment, 10 ng of the pEASY-GFP plasmid was used as the template with modifications of perfectly matched forward and reverse primers to create a series of mismatched primers according to Ayyadevara et al. [27] from the symmetry axis to the 3’ end (**S1 Table**).

### Quantitative real-time PCR reactions

Real-time PCR amplification was performed using Bio-Rad SYBR Green I Master according to the manufacturer’s instructions; 500 nM primers and a variable amount of DNA standard were used in a 20 μl final reaction volume. Amplification was performed in a programmable thermal cycler (Bio-Rad 480, USA) under the following conditions: after 10 min of denaturation at 95 ^o^C, 40 amplification cycles (95°C for 5 s; 60°C for 5 s; 72°C for 20 s) were performed with a single fluorescence reading taken at the end of each cycle. For the long amplicon, the elongation was at 72°C for 90 s. Each reaction combination was performed with three replicates, and all the runs were completed with a subsequent melt curve analysis. The PCR product was resolved on ethidium-bromide-stained 1% agarose gel to confirm the specificity of bands and absence of primer dimers.

### Outlier cycle determination

**Fig 1:**
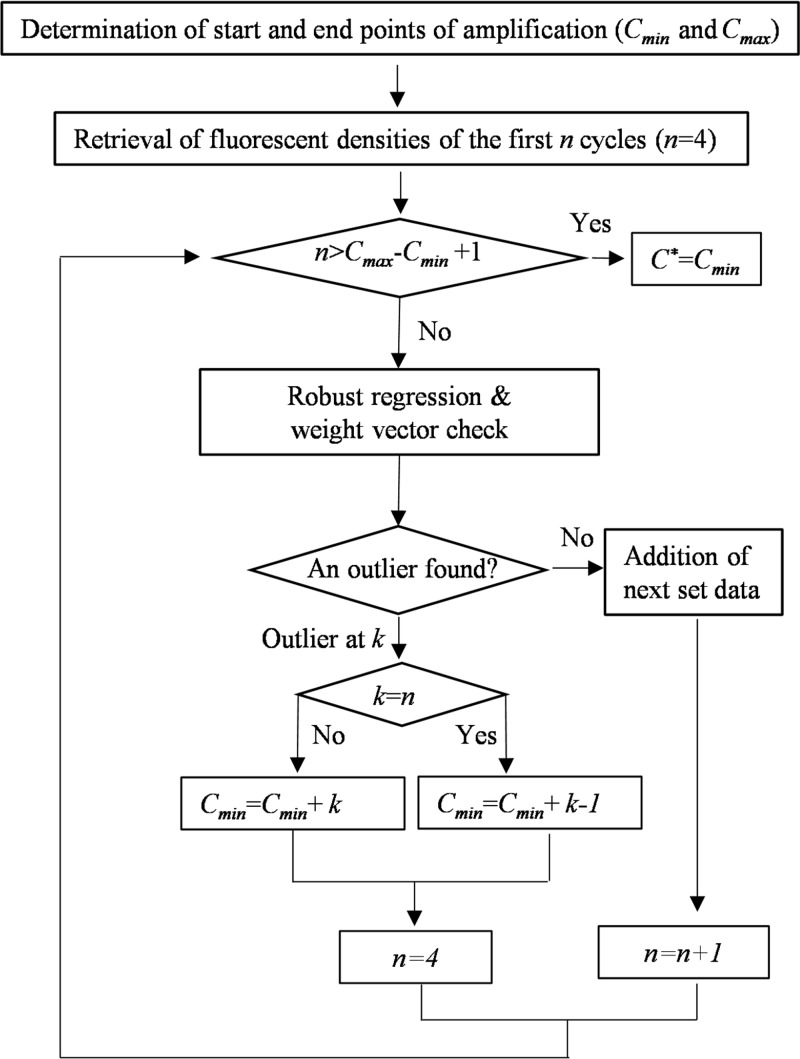
A flowchart for determination of the outlier cycle C* based on robust regression analysis. The process was started by determining the minimal and maximal amplification points *C*_min_ and *C*_max_ using retrieved logarithmic data of fluorescence intensities from a PCR reaction; this was followed by identification of the outlier cycle C* located within *C*_min_ and *C*_max_ by robust regression analysis. This process would be conducted with the first *n* (*n* = 4) observations. In the case that no outlier was found in these *n* points by checking weight vector of robust regression, another observation would be added for a further round of checking until the outlier cycle was identified.

As a q-PCR amplification process starts, the fluorescence intensity of the amplified products integrated with dye molecules increases; the cycle at which the product fluorescence is strong enough to be differentiated statistically from the background is defined as the outlier cycle ($C^{*}$), and the method to obtain it is depicted in **Fig 1**according to Viechtbauer and Cheung [28]. For a given set of qPCR data retrieved from Bio-Rad CFX Manager, the averages of fluorescence intensities were obtained for every cycle from technical triplicates. From these data, we identified the minimum amplification cycle ($C_{\min}$) at which the fluorescence intensity was the lowest but positive (>0) and the amplification efficiency ($E_{i}=\frac{F_{i+1}}{F_{i}}$) ranged between 0.2 and 1.1, largely due to background variations. Also determined by inflection point identification was the maximum amplification cycle ($C_{\max}$), where the maximal difference in fluorescence intensity was found between two consecutive cycles ($\Delta F_{i}=F_{i+1}-F_{i}$). The fluorescence intensities from cycle $C_{\min}$ to cycle $C_{\max}$ were then converted to logarithmic data for further robust regression analysis.

Since the fluorescence of the amplified product increases exponentially at the beginning phase, a linear relationship between the cycle and the log of the fluorescence intensity exists theoretically as the following:

$$log(F_{C})=a+b\cdot C$$ (2)

where $a=log(F_{0})$, b=log(1+E), and *C* is the completed cycle number. By following Eq 2, the outlier of the amplification cycle ($C^{*}$) from $C_{\min}$ to $C_{\max}$ could be identified based on the observed fluorescence intensities by robust linear regression analysis [28]. The data of the first set of four consecutive cycles (*n* = 4) was analyzed by checking weight vector of robust regression. In the case that no outlier in these *n* tested points was found, additional next cycle data (*n* = *n*+1) were included for further regression analysis until $C_{\max}$ was reached. When an outlier was identified at the *k*^th^ cycle within the tested *n* points, a new $C_{\min}$ was re-defined as the following:

$$C_{\min}=\{\begin{array}{ll} C_{\min}+k-1, & k=n\\ C_{\min}+k, & k\neq n \end{array}$$ (3)

Then another round of robust regression analysis was performed starting from the newly defined $C_{\min}$. The starting point $C_{\min}$of the last robust regression analysis was identified as the outlier ($C^{*}$).

### Generation of simulation data with scalar values for further verification

In order to further evaluate the new data processing approach, a statistical simulation test was performed to address the estimation of the outlier cycle and the initial fluorescence intensity. A simulation was tested over 2000 times. $F_{max}$ and $E_{max}$ were arbitrarily set as the respective constants 4000 and 0.7 based on the ranges of fluorescence intensities and amplification efficiencies obtained from our regular PCR data. The outlier cycles were also arbitrarily set at cycles 5, 10, 15, 20 and 25, and their corresponding initial fluorescence intensities were set at 5, 0.5, 0.05, 0.005, and 0.0005 (**S2–S6 Tables**). In addition, over 2000 simulation tests were performed with different random disturbances integrated into the fluorescence intensity curves according to Eq 4. Within a section starting from Cycle 0 to Cycle C*-1, random variation from $-\frac{F_{C*}}{2}$ to $\frac{F_{C*}}{2}$ (half of the fluorescence intensity at C*) was applied to each cycle. Within the section beyond, the variation was applied with a random disturbance of ± 2 at each cycle [29].

$$F=\frac{F_{\max}}{1+(\frac{F_{\max}}{F_{0}}-1){\left(1-E_{\max}\right)}^{-C}}$$ (4)

where $F_{\max}$ is the maximal fluorescence intensity and $E_{\max}$ is the maximal efficiency of the amplification curve. Consequently, simulation data were generated for further evaluation of the new data processing approach.

### Performance evaluation

In order to evaluate the performance of CyC*, the public data of qPCR reactions for 63 genes (excluding AluSq) developed by Vermeulen et al.[30] (http://www.dr-spiess.de/qpcR/datasets.html) were retrieved and used for calculation of performance indicators bias, linearity, reproducibility, and resolution according to Ruijter et al. [31] and Bultmann and Weiskirchen [32]. The resulting indicators of CyC* were compared with corresponding indicators values of all other 13 methods including *Cy0*, Standart_Cq, and LinRegPCR [31]. To examine the effects of the initial DNA level on the performance of different methods, the performance indicators of all tested methods were compared with the data generated at the highest three levels of DNA input separately, in addition to the comparison conducted with the data at all five DNA input levels.

### Validation of the new method

cDNA obtained from tea plants was employed for new method validation. Two 1 μL cDNA template samples were applied in qPCR reaction mixtures, with one at the original concentration and the other at a 5-fold dilution; qPCR was performed with the same pairs of gene specific primers (**S1 Table**). The qPCR program was coded, compiled and performed as described above. After verification of gene amplification through melting curve analysis (**S1 Fig**), the raw data were retrieved and analyzed using CyC* and compared with 2^-ΔΔCt^ method. Gene 1 (18s rRNA, GenBank, AY563528.1) and Gene 2 (CSA008212.1) from tea genome information [33]were employed as reference genes for relative expression analyses between data using two different starting cDNA amounts. The relative gene expression levels and their confidence intervals at 99% probability were plotted for all tested genes.

## Results

### Occurrence of cumulative error

The amount of PCR amplified product intercalated with fluorescent reporter molecules in any amplification cycle could be presented as the fluorescence intensity of the amplified DNA ($S_{C}$) modified with a constant product-specific coefficient (α) in Eqs 5 and 6 [2]:

$$F_{C}=\alpha S_{C}$$ (5)

$$F_{C}=F_{C-1}(1+E_{C})$$ (6)

Where *F*_*C*_ and *E*_*C*_ are the fluorescence intensity and amplification efficiency at the reaction cycle *C*, respectively, and $F_{C-1}$ is the fluorescence intensity at the previous cycle. It follows that PCR efficiency at any given amplification cycle *C* can be represented by Eq 7:

$$\frac{F_{C}}{F_{C-1}}=1+E_{C}$$ (7)

Because *E*_*C*_ changes from cycle to cycle and is rarely ideal (100%), the error in the amplified product amount quantification, named as $\Delta F_{C}(C=1,2,3,\cdots )$, occurs in each cycle between the real $F_{C}$ value and the theoretical value, which would be often calculated based on assumed perfect amplification efficiency methods. $\Delta F_{C}(C=1,2,3,\cdots )$ will be augmented continuously as Eq 8 describes:

$$\{\begin{array}{l} \Delta F_{C}=F_{C-1}\cdot (1-E_{C})+\sum_{i=1}^{C-1}\Delta F_{i}\\ \Delta F_{1}=F_{0}\cdot (1-E_{1}) \end{array}$$ (8)

Then, the cumulative error from cycle 0 to cycle k+1, represented as $\varepsilon_{k+\text{1}}$, should be bigger than the error $\varepsilon_{k}$ at cycle *k* and it follows Eq 9:

$$\varepsilon_{k+1}=\sum_{C=1}^{k+1}\Delta F_{C}=\varepsilon_{k}+\Delta F_{k+1}>\varepsilon_{k}$$ (9)

**Fig 2:**
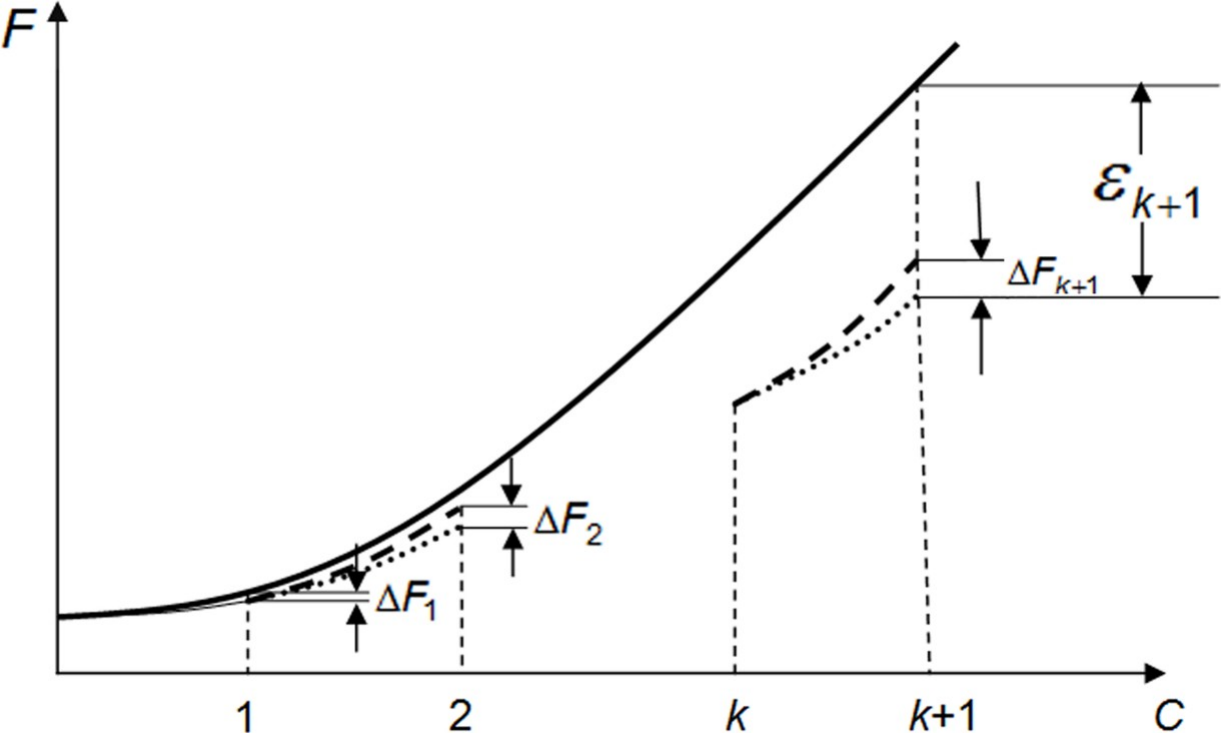
The relationship between the cumulative error and error in each step. In the plot, a solid line represents theoretical curve of amplified product ($F_{c}$) with $E_{C}\equiv 1$; a dashed line represents theoretical curve of amplified product with $E_{C}\equiv 1$; a dotted line represents the obtained curve of amplified product with ${Ε}_{C}<1$. Δ*F*_1_ and Δ*F*_2_ were the differences between real measurements and theoretical values after the first and second cycles, respectively. $\varepsilon_{k+1}$ represents the cumulative error starting from cycle 0 to cycle *k+*1.

Accordingly, $\Delta F_{2}$ is the error accumulated from the first two cycles and $\Delta F_{1}$ is the error after the first cycle (**Fig 2**). Obviously, $\Delta F_{2}$ is bigger than $\Delta F_{1}$, indicating that the cumulative error increases as the amplification reaction proceeds and that the amplified product quantification should be performed in the earliest cycle as possible to minimize the cumulative error.

### Outlier cycle determination

In a regular qPCR, background fluorescence makes it difficult to determine the beginning of the actual amplification cycle. However, since the fluorescence of the amplified product at the beginning phase increases exponentially, a linear relationship between the cycle and the log of the fluorescence intensity exists as Eq 2. The outlier of the amplification cycle ($C^{*}$) could be identified by Eq 2 based on the observed fluorescence intensities from *C*_min_ to *C*_max_ by robust linear regression analysis as previously described [28]. If no outliers were found in these tested points (*n*), additional data of next cycle (*n*+1) were included for regression analysis (weight vector of robust regression) until it was found. When the outlier was identified in any cycle of *n-1* points, the cycle immediately after the identified outlier was reset as a new starting cycle ($C_{\min}$) for a new round of robust regression analysis to find possible another outlier till the inflection point reached. However, in case that Cycle *n* was identified as the outlier, Cycle *n* was reset as $C_{\min}$to continue the search for C*.

### Estimation of amplification efficiency and initial fluorescence intensity

At the first several amplification cycles, the detected fluorescence intensity could hardly be doubled, due to the background interference and limited signal detect threshold. Based on the public PCR datasets <http://www.dr-spiess.de/qpcR/datasets.html.> provided by Ruijter et al. [34], the random error $\varepsilon_{c}$ of the fluorescent intensity of the amplified product linearly correlated to the amplification cycles. Considering this error, the initial fluorescence intensity ($F_{0}$) could be obtained based on the detected intensity of fluorescence ($F_{C*}$) and the error $\varepsilon_{c^{*}}$ at the outlier ($C^{*}$) cycle as Eq 10. Since a linear relationship between the cycle and the log of the fluorescence intensity exists as per Eq 2, the amplification efficiency at C* could be obtained by fitting the four consecutive points from the outlier ($C^{*}$) with linear regression as per Eq 11.

$$F_{0}=\frac{F_{c*}-\varepsilon_{c^{*}}}{2^{c*}}$$ (10)

E=exp(b)−1 (11)

where $\varepsilon_{c^{*}}$ is the random error of the fluorescence intensity at $C^{*}$, *b* is the slope of the fitted line of converted logarithmic fluorescence intensities. The fluorescence intensity of the initial DNA template was obtained with minimal cumulative error. For application of this method, a computer program “CyC* method” was compiled using MATLAB software, which can be downloaded as a supplementary file. Moreover, if the conversion coefficient of the DNA template to fluorescence is determined as α [35], the initial amount of the template DNA segment can be obtained using Eq 12.

$$S_{0}=\frac{1}{\alpha }F_{0}$$ (12)

In practice, the amounts of the initial target DNA templates in samples could be quantified once α is obtained using a known amount of template input derived from artificially synthesized oligo or a plasmid containing the target DNA segment. For the relative quantitative analysis of a target gene expression, the ratios of transcript levels of a target gene over the corresponding transcript levels of the stably expressed reference gene in different samples can be obtained, and the expression fold changes of the target gene in different samples can be calculated according to Eq 13.

$$\text{Fold Change}=\frac{\omega_{1}}{\omega_{2}},\omega_{i}=\frac{F_{i}^{ot}}{F_{i}^{or}}\;(i=1,2)$$ (13)

where $F_{i}^{ot}$ and $F_{i}^{or}$are the calculated initial fluorescence intensities *F*_0_ respectively for the target and reference genes in the sample *i*.

### Validation with simulation and qPCR tests

**Fig 3:**
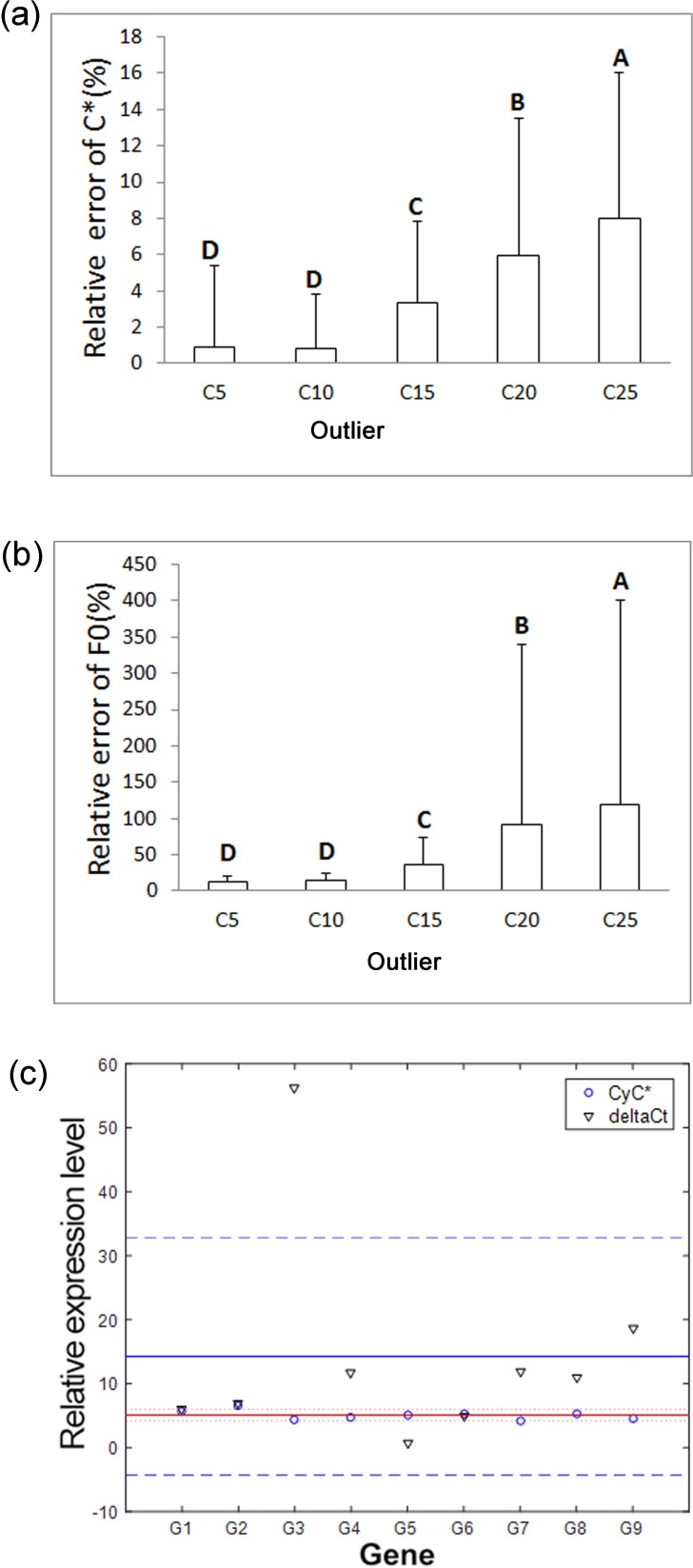
Verification of the CyC* data processing approch using simulations and actual qPCR tests. (a), Relative errors of the outliers (C^*^) for 5 different outliers obtained using C^*^ and estimated C^*^ based on the robust regression analyses in 2000 simulations. Columns with different capital letters differ significantly by one-way analysis of variance (*p* <0.05); (b) Relative errors of the initial fluorescence intensity (*F*_0_) for 5 different outliers obtained using *F*_0_ and estimated ${\overset{\wedge }{F}}_{0}$ based on linear regression analyses in 2000 simulations. (c) Plot of relative expression levels of all nine tested nine terpenoid synthase genes (G1-G9) with their confidence intervals at 99% probability obtained by CyC* and 2^-ΔΔCt^ methods using two initial amounts (5:1) of the same cDNA template with the expected 5-fold difference between the two. The abnormal level obtained for G3 using the 2^-ΔΔCt^ methods was ignored when two methods were compared.

Theoretically, the fluorescence intensities of qPCR conform to the logistic growth mode as per Eq 4 [29]. For verification of this new approach, a simulation test was conducted for 2000 times according to Eq 9 using fixed *F*_max_, *E*_max_, but varying the outlier cycles ($C^{*}$) with inversely proportional variations of their corresponding initial fluorescence intensities as specified in the part of Methods. Our simulation results revealed that the outlier cycles and initial fluorescence intensities were all close to the actual values (**Table 1**) with small standard deviations. The one-way analysis of variance revealed that both the relative errors of the outlier cycles and the initial fluorescence intensity (F0) increased significantly (*p* < 0.05) with delayed appearance of the outlier cycles (**Fig 3A and 3B**) (**Table 1**). Our simulation test indicated that advancing the emergence of the outlier cycle can significantly reduce the estimation error of *F*_0_ and C*.

**Table 1:** The means and variances for estimation of different parameters in 2000 simulations.

Outlier	Parameters	Actual value	Mean_pre	Se_pre
5	*First_outlier*	5	4.953	0.2209421
	*F*_*0*_	5	5.271170095	0.6751955
10	*First_outlier*	10	9.9205	0.3003745
	*F*_*0*_	0.5	0.440933114	0.0688352
15	*First_outlier*	15	14.499	0.6769255
	*F*_*0*_	0.05	0.039339932	0.0238827
20	*First_outlier*	20	18.8135	1.5082623
	*F*_*0*_	0.005	0.001278754	0.0126218
25	*First_outlier*	25	23.0105000	2.0135583
	*F*_*0*_	0.0005	8.61427E-05	0.0014755

Our simulation test revealed that variations in initial fluorescence intensities (*F*_*0*_) had greater effects than those for the outlier ($C^{*}$). suggesting that the outlier cycle identified in this study was quite reliable. likely because the random variation relative to the fluorescence intensity set in the test was consistent through all cycles, such that variations were greater relative to the low fluorescence intensities during cycles around the outlier than variations during late amplification stages with high fluorescence intensities. In order to minimize the variation of fluorescence intensities, initial template amounts should be increased.

Moreover, a set of regular qPCR was performed to quantify the transcript levels of nine genes (represented by G1 to G9), which putatively encode terpenoid synthases; the same cDNA templates were each used at two concentrations with a ratio of 5:1. The melting curves of the amplified products with single peaks for each gene (**S1 Fig**) suggested specific amplification. The relative transcript levels for each of the genes were calculated for the two reactions using CyC* and compared with the data obtained using the 2^-ΔΔCt^ method (**Fig 3C**); tea *18s rRNA* and Gene 2 were used as reference genes. Theoretically the relative transcript ratios of all the tested genes in the non-diluted over the diluted cDNA templates should be 5. The average ratio and standard deviation obtained with CyC* for all tested genes was 5.11±0.70, which was closer to 5 than the average ratio of 7.79 ± 4.62 obtained with the 2^-ΔΔCt^ method after removal of abnormal data from Gene 3. Our data suggested that CyC* method resulted in more accurate results than the conventional method, which resulted from minimization of cumulative error.

### Performance evaluation

Performance comparison between CyC* and other 13 qPCR analysis methods was conducted using public data of qPCR reactions [30]. For each method, the values the performance indicators bias, linearity, reproducibility, and resolution were calculated for all five or for the highest three DNA input levels (**S7–S16 Tables** representing *F*_*0*_ and C* estimates as well as the values of four indicators at two sets of DNA input). The mean rank of all these indicators was obtained for each of these methods. The sorted methods based on the mean rank were further statistically analyzed with the Friedman test. In case of the highest three DNA input levels, CyC*, grouped with MAKERGAUL_C, Standart_Cq, LinRegPCR, and PCR_Miner was ranked after *Cy0* (**Table 2**). Once all the five DNA input levels were considered, CyC*, together with other four methods, was ranked after *Cy0*, LinRegPCR, and Standart-Cq. These data indicated that the overall performance of CyC* was better than majority of the tested methods. The initial DNA put levels affected CyC* performance.

**Table 2:** Comparative performance analysis of CyC* using public datasets.

Method	Bias	Linearity	Reproduci-bility	Resolution	Mean rank	Friedman test subset						
With the first three highest DNA input levels												
Cy0	2.68(1)	2.97(1)	5.21(3.5)	3.16(1)	1.625	1						
MAKERGAUL_C	5.86(3)	6.27(4)	5.21(4.5)	5.49(5)	3.875	1	2					
Standart_Cq	3.17(2)	4.14(3)	5.48(7)	4.83(4)	4	1	2					
CyC*	6.21(4)	7.48(5)	4.57(2)	5.83(7)	4.5		2	3				
LinRegPCR	8.05(8)	7.54(6)	3.84(1)	4.76(3)	4.5		2	3				
PCR_Miner	8.49(10)	3.52(2)	5.24(5)	3.79(2)	4.75		2	3				
MAK2	7.16(7)	7.73(8)	5.29(6)	5.68(6)	6.75			3	4			
LRE_E100	7.02(6)	7.56(7)	5.95(9)	6.25(8)	7.5				4			
MAKERGAUL	6.56(5)	8.10(10)	5.83(8)	6.76(9)	8				4	5		
5PSM	10.22(13)	7.83(9)	9.62(10)	9.35(10)	10.5					5	6	
4PLM	8.19(9)	9.30(11)	11.94(11)	11.89(12)	10.75						6	
DART	9.67(11)	9.46(12)	11.98(12)	11.67(11)	11.5						6	7
LRE_Emax	9.68(12)	11.16(13)	12.37(13)	12.59(13)	12.75						6	7
FPK_PCR	12.05(14)	11.95(14)	12.49(14)	12.95(14)	14							7
With all five DNA input levels												
Cy0	2.25(2)	3.46(1)	4.05(2)	3.63(2)	1.75	1					
LinRegPCR	7.11(5)	4.17(2)	2.68(1)	2.78(1)	2.25	1					
Standart-Cq	2.21(1)	4.90(3)	4.63(3)	4.54(3)	2.5	1					
MAKERGAUL_C	6.73(3)	5.54(5)	6.24(6)	5.79(5)	4.75		2					
PCR-Miner	8.92(10)	5.27(4)	5.52(4)	5.22(4)	5.5		2	3			
MAK2	7.94(7)	5.98(6)	6.16(5)	6.06(6)	6		2	3			
CyC*	6.98(4)	6.70(8)	6.52(7)	6.54(7)	6.5		2	3			
LRE-E100	8.21(8)	6.17(7)	6.67(8)	6.60(8)	7.75			3	4		
MAKERGAUL	7.38(6)	6.98(9)	6.68(9)	6.71(9)	8.25				4		
5PSM	9.71(12)	8.60(10)	8.41(10)	8.79(10)	10.5					5	
DART	9.30(11)	10.65(11)	10.68(11)	11.03(11)	11					5	
4PLM	8.52(9)	11.38(12)	11.67(12)	11.54(12)	11.25					5	
LRE-Emax	9.95(14)	12.03(13)	12.40(13)	12.46(13)	13.25						6
FPK-PCR	9.78(13)	13.14(14)	12.68(14)	13.29(14)	13.75						6	

### Emergence of the outlier affected by initial DNA template amounts

**Fig 4:**
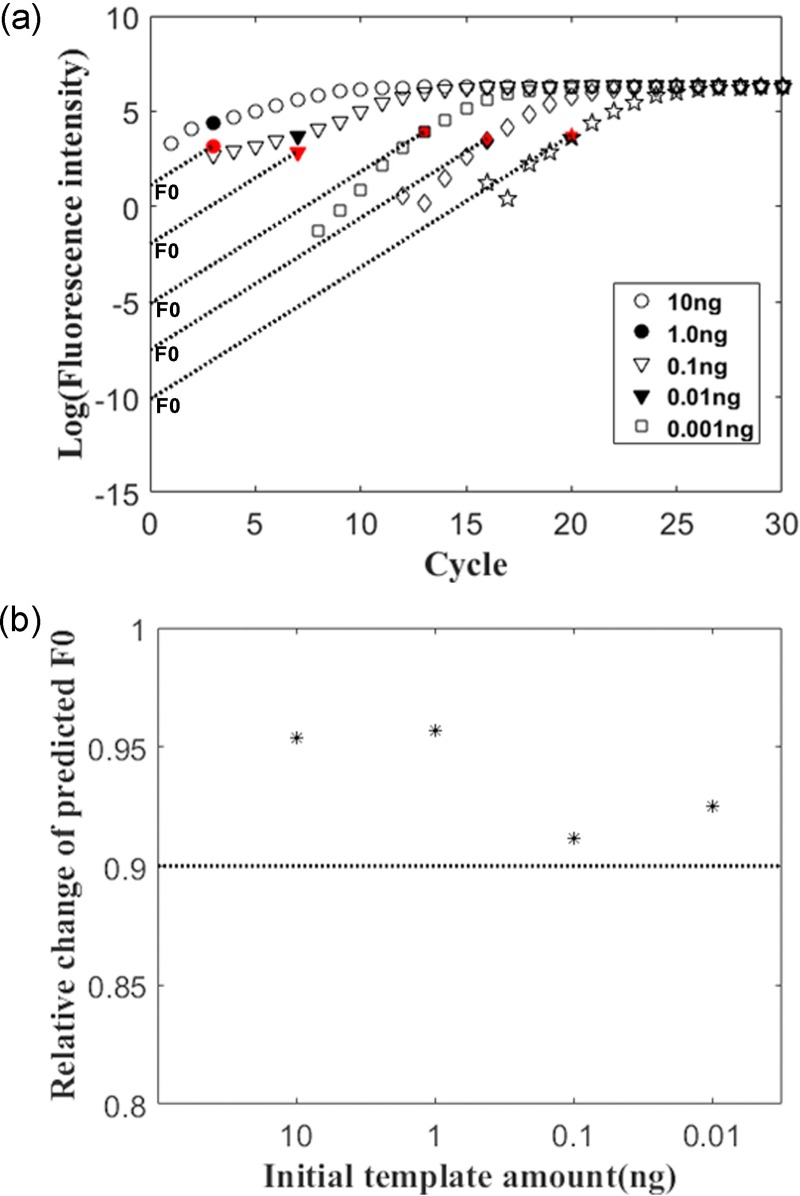
Effect of initial template amount on emergence of the outlier and predicted F_0_ accuracy. (a) Outlier emergence (black spots in the amplification curves) and its adjusted outlier fluorescence intensities (Red spots) were affected by different initial amounts of DNA template. The intersections of lines with the Y axis were the predicted fluorescence intensities *F*_0_ with minimum cumulative errors; (b) Effects of initial DNA template amount on relative changes in the predicted *F*_0_ values. The dashed line refers to theoretic relative change of *F*_0_ values at one level of the template DNA relative to the 10-fold diluted level.

qPCR reactions were performed with different amounts of initial template DNA to examine the effect of template amount on emergence of the outlier cycle. Our data indicated that the emergence of the outlier of the amplification curve was dependent on the initial amount of DNA template. The outlier appeared at the third cycle when the initial DNA input was 10 ng, which was earlier than the outliers of other reactions with lower levels of template DNA. Correspondingly, the predicted initial fluorescence intensities using CyC* method varied (**Fig 4A**). However, when a series of 10-fold diluted DNA templates were employed, the relative change in the predicted *F*_0_ using two 10-fold dilutions with high levels of the templates was much closer to 0.9 compared to the predicted *F*_0_ values of 10-fold dilutions with low levels of the templates (**Fig 4B)**. The relative change in *F*_*0*_ between the one initial template level and its 10-fold diluted one was expected to be 0.9 in the case that values for the qPCR amplified product fluorescence conversion coefficient α were the same. Our results indicated that an increased level of the initial amount of DNA template within the tested range was effective in advancing outlier emergence, reducing cumulative error, and thereby improving quantitative results.

### Effect of amplicon sizes on the outlier emergence

**Fig 5:**
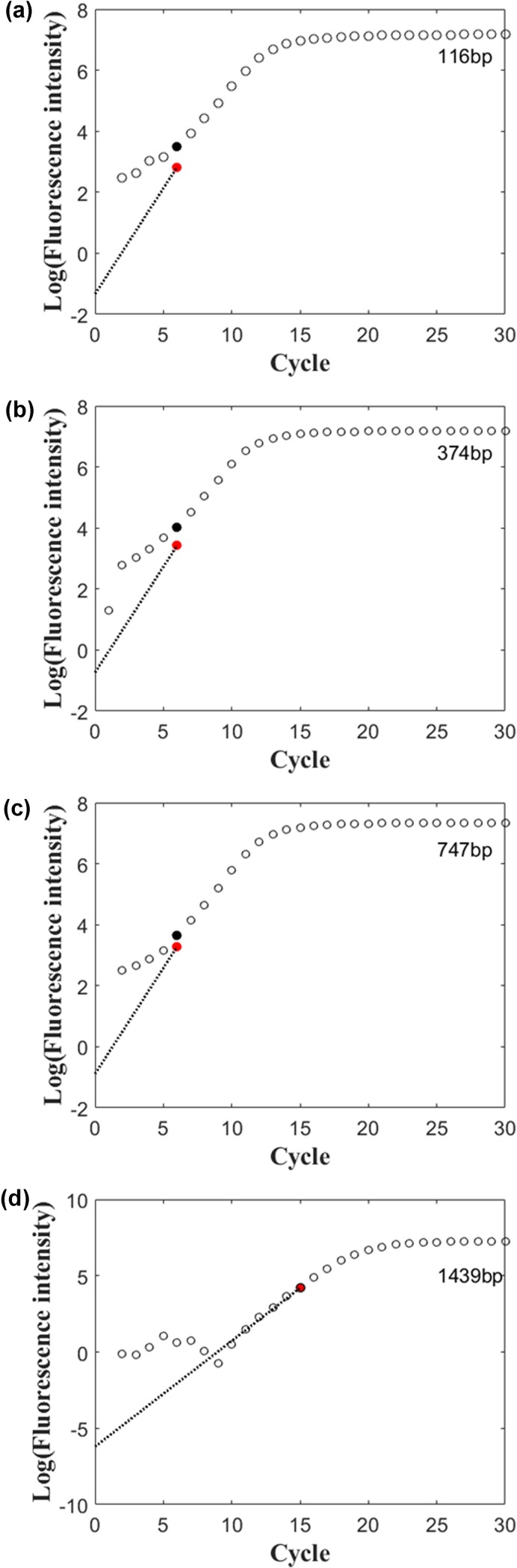
Effect of amplified product sizes (116 bp, 347 bp, 747 bp, 1439 bp) on emergence of the outlier. Black and red dots represent the intensities of detected and adjusted fluorescence signals at outliers in the amplification curves.

To test the effect of product size on the emergence of the outlier cycle, primer pairs were designed to produce a series of PCR products with increased sizes (116, 347, 747 and 1439 bp). Our data indicated that the outliers appeared at the 6^th^ cycle with three smaller tested products **(Fig 5)**. For the largest product (1439 bp), the outlier emergence was delayed to 15^th^ cycle. An adequately size of the amplified PCR products could possibly advance the appearance of outliers, and thereby reduce cumulative error.

### Effect of primer mismatch on emergence of the outlier

**Fig 6:**
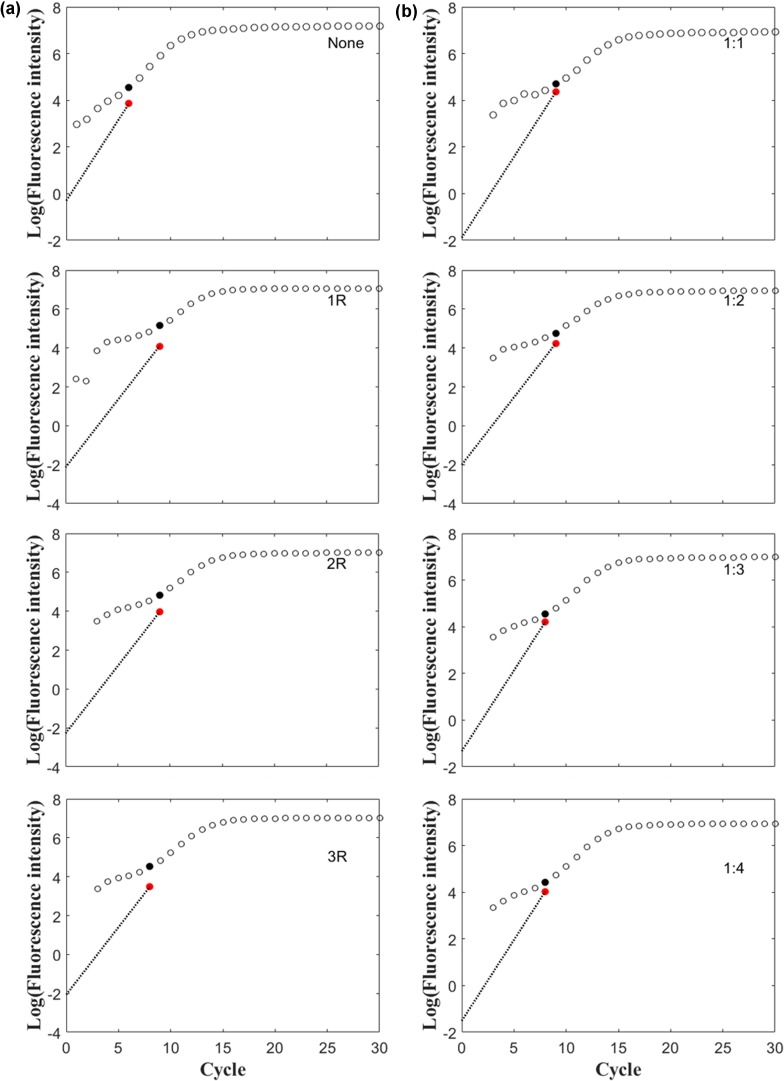
Effects of different combinations of matched and mismatched primers on outlier emergence. (a), PCR amplification with changed position of the outlier cycle using a pair of primers both with two mismatched nucleotides located at the 9^th^ and 10^th^ (middle position), 12^th,^ and 13^th^, 13^th^ and 14^th^ nucleitides represented by 1R, 2R, and 3R, respectively. (b), Ratios of mismatched primers to the non-mismatched primers represented by 1:1, 1:2, 1:3, and 1:4, respectively.

The primer-template mismatch and its location could strongly decrease amplification efficiency [18]. In this study, different mismatch location in primers and different combinations of mismatched primers were compared with perfectly matched primers (**S1 Table**) to determine the effects of primer-template mismatches on outlier appearance. Our data indicated that the mismatched template-primer delayed the outlier emergence than the non-mismatch primer **(Fig 6A)**. However, the mismatched locations tested in this study did not result in a dramatic shift of the outlier emergence. Moreover, outlier emergence was advanced with declines in the ratio of mismatched primers over the correct primers **(Fig 6B)**. These data indicated that primer-template mismatches in qPCR analysis should be avoided in order to advance the emergence of the outlier.

## Discussion

In this study, the cumulative error in the qPCR process was revealed by kinetics analysis. Our study indicated that this error in the quantification of initial template amount could increase as amplification proceeded; this was due to both the difference in the actual template amount and estimates which were based on the assumption of perfect amplification efficiency. The conventional and widely employed threshold cycle (*Ct*) method requires assumed perfect amplification efficiency [13], which rarely occurs in practice [29,33]; this consequently leads to an inaccurate estimate of initial template amounts of both the genes of interest and of reference genes. To avoid amplification efficiency assumption, many investigations have been carried out to develop new approaches for transcript analysis. For example, the sigmoid curve fitting method (SCF) was developed to fit the sigmoid model so that the initial template amount can be deduced from the fluorescence (*F*_0_) without the need for standard curve [36]. BestKeeper was reported to determine transcript stability of tested gene using pair-wise correlations [25]. Another linear regression method, taking-difference between consecutive two cycles was recently developed to remove the background fluorescence interference [24]. However, the majority of previously reported data processing methods have largely ignored cumulative error.

In this study, the novel CyC* data processing method, was proposed to minimize cumulative error by identifying and advancing the outlier cycle for the purpose of improving qPCR quantitative analysis. Performance comparison revealed that CyC* exhibited a higher ranking than majority of the 13 tested methods, only after *Cy0*, or LinRegPCR, and Standart-Cq, depending on DNA input levels. Nevertheless, *Cy0* and Standard-Cq require calibration in every real time PCR experiment standard curves which have to be prepared [16,31]. LinRegPCR demands to determine the individual PCR efficiency of every sample[31,37]. On the contrary, CyC* does not have these requirements and easy to perform, particularly using the computer program “CyC* method” for data processing attached to this report. Moreover, simulation and actual PCR tests indicated that this method produced improved results over the conventional 2^-ΔΔCt^ method in relative quantitative analyses. Once the coefficient α is obtained for the conversion of a specific DNA segment to fluorescence intensity [35] in a fixed qPCR system, this method could be used for absolute quantification of initial DNA template amounts as well.

In the CyC* approach, one of the critical steps is to determine and advance the outlier cycle as early as possible to minimize cumulative error. Combined with practical manipulation of several qPCR conditions such as initial template amount and product size, the outlier emergence could be advanced to different extents, which further reduced cumulative error. It is reasonable to see that effective outlier advancement took place simply by increasing the initial template amount from 0.1 ng to 10 ng in this study. In many cases such a template augment can be applied without significantly increased costs. Moreover, increased DNA input can advance the outlier cycle, consequently, the PCR reaction could be stopped much earlier than usual (i.e. 40 cycles). In this way, both PCR reaction components and time can be substantially reduced. Moreover, with the increased template input, CyC* could be able to detect the relative difference in initial template abundance at low level with improved accuracy. However, amplification inhibition could occur due to increased template competition or contaminant inhibitors once the initial DNA input is increased significantly. To avoid such an inhibition, DNA template should be carefully prepared to minimize protein and other chemical contaminants with commercial RNA or DNA extract kits and DNA input should be adequate.

Moreover, product size can be easily manipulated by priming site selection at the primer design stage. It has been shown that small size of amplicon around 100 bp could be amplified with high amplification efficiency [38]; thus, small amplicons are widely accepted for qPCR analysis. It was speculated that a single small amplicon molecule generally has less fluorescence intensity than a larger amplicon molecule when the same fluorescent dye is used for intercalation. However, in this study the outlier cycles in the PCR reaction resulting in PCR product sizes ranging from 116 bp to 747 bp all emerged in Cycle 6 and the outlier of the PCR reaction generating product with 1439 bp delayed; this could be due to significant decreases in amplification efficiency with larger amplicon sizes as previous reported [38]. Our data indicated that the inappropriately increased amplicon size could negatively affect performance of quantitative PCR analysis. In case that fluorescent probes rather intercalating fluorophores are used for fluorescence quantification, effect of the amplified product sizes on outlier emergence might be very limited.

In this study, effect of mismatches between primer and target DNA on outlier appearance time of was also examined. Our data revealed that primer mismatches and decreased ratio of non-mismatched primers over mismatched primers led to a substantial delay of outlier cycle appearance. Our results were consistent with previous reports that template-primer mismatches can affect amplification efficiency and hamper the ability to amplify the target DNA [39]. It has been shown that single primer-template mismatches at the 3’ end of the primer sequence can prevent PCR [40]. Increasingly negative effects on amplification efficiency have been observed in other studies with mismatch sites closer to the 3’ end of the primer [27,41]. Although mismatches between template and primers can be avoided at the primer design stage, the ratio of correct primer molecules over the mismatched primer ones could have an effect due to interference by partially matched cDNA molecules from the reverse transcribed cDNA background; thus, careful checking should be conducted to avoid possible mismatches of the designed primers with the transcriptomic background of the target plant species.

### Conclusions

Our study revealed the existence of cumulative error in the qPCR data processing methods which require the assumption of perfect amplification efficiency. The CyC* method is based on determining the emergence of the outlier related to actual amplification efficiency to minimize cumulative error. Software has been developed for this data processing method, and it has been validated with simulations and actual qPCR tests. Increased initial template amount in a PCR reaction and appropriate amplicon size were able to advance the emergence of the outlier for improvement in the accuracy of quantitative analyses.

## Supporting information

S1 Fig Melting curve verification of the gene amplification. (TIF)

S1 Table Primer pairs used in this study. (XLSX)

S2 Table The predicted values and the relative errors for two different parameters in each simulation (The true value of first outlier cycle was 5, and the ture value of initial fluorescent intensity was 5). (XLSX)

S3 Table The predicted values and the relative errors for 2 different parameters in each simulation (The true value of first outlier cycle was 10, and the true value of initial fluorescent intensity was 0.5). (XLSX)

S4 Table The predicted values and the relative errors for 2 different parameters in each simulation (The true value of first outlier cycle was 15, and the true value of initial fluorescent intensity was 0.05). (XLSX)

S5 Table The predicted values and the relative errors for 2 different parameters in each simulation (The true value of first outlier cycle was 20, and the true value of initial fluorescent intensity was 0.005). (XLSX)

S6 Table The predicted values and the relative errors for 2 different parameters in each simulation (The true value of first outlier cycle was 25, and the true value of initial fluorescent intensity was 0.0005). (XLSX)

S7 Table Estimate values of F_0_ using CyC* and other 13 published methods with publicly available data. (XLS)

S8 Table Estimate values of *Cq* using CyC* and other 13 published methods with publicly available data. (XLSX)

S9 Table Bias comparison between CyC* and other 13 published methods using three highest concentrations of DNA inputs. (XLSX)

S10 Table Linearity comparison between CyC* and other 13 published methods using three highest concentrations of DNA inputs. (XLSX)

S11 Table Reproducibility comparison between CyC* and other 13 published methods using three highest concentrations of DNA inputs. (XLSX)

S12 Table Resolution comparison between CyC* and other 13 published methods using three highest concentrations of DNA inputs. (XLSX)

S13 Table Bias comparison between CyC* and other 13 published methods using all five concentrations of DNA inputs. (XLSX)

S14 Table Linearity comparison between CyC* and other13 published methods using all five concentrations of DNA inputs. (XLSX)

S15 Table Reproducibility comparison between CyC* and other 13 published methods using all five concentrations of DNA inputs. (XLSX)

S16 Table Resolution comparison between CyC* and other 13 published methods using all five concentrations of DNA inputs. (XLSX)

## Abbreviations

Ct: Cycle-threshold
qPCR: quantitative real-time polymerase chain reaction
*E*: efficiency
GFP: Green fluorescent protein
SCF: Sigmoid curve fitting method
SOD: Shape based kinetic outlier detection
