# Supplementary figures and images for: A novel data processing method CyC* for quantitative real time polymerase chain reaction minimizes cumulative error
Source: PLoS One. 2019 Jun 11;14(6):e0218159. doi: 10.1371/journal.pone.0218159 (PMC6559663; doi:10.1371/journal.pone.0218159)

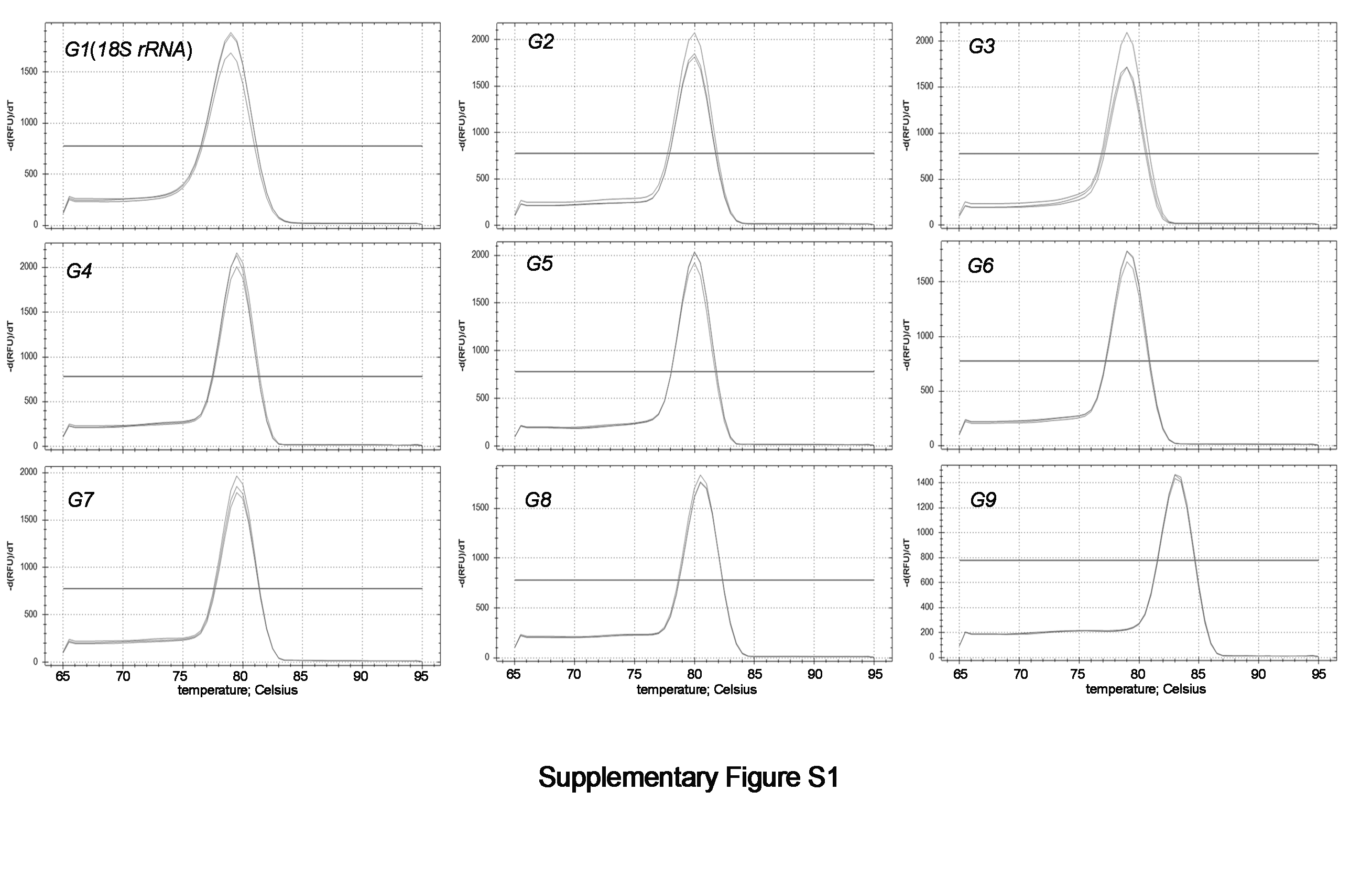

Supplement: S1 Fig — (TIF) [file pone.0218159.s001.tif]
